# Comment on “Short-Term Efficacy of Ultramicronized Palmitoylethanolamide in Peripheral Neuropathic Pain”

**DOI:** 10.1155/2014/849689

**Published:** 2014-11-27

**Authors:** R. Kriek

**Affiliations:** JP Russell Science Ltd, 30 Chytron Street, Office A32, 1075 Nicosia, Cyprus

In the recent paper published [1] in this journal, the efficacy and safety of a specific palmitoylethanolamide were reported: ultramicronized palmitoylethanolamide (PEA-um). This paper is one of recent series trying to convince readers that only a specific and patented formulation produced by the company Epitech Group S.r.L. in Italy is effective and has been explored sufficiently. This claim is not supported by the available evidence. We have discussed grave misrepresentations of data in a different paper [2]. We highlighted in that paper that the group of Professor Cuzzocrea repeatedly presented scientific facts related to unformulated palmitoylethanolamide (PEA) as if the data originated from experiments with ultramicronized PEA.

The paper we discuss here is therefore not the first to wrongly attribute efficacy data gathered by pure, unmicronized palmitoylethanolamide to the so-called PEA-um formulation. For instance, the authors state on p. 1 the following:* “PEA-um demonstrated a significant efficacy on pain in the murine model of diabetic neuropathy”* and they refer to the data on palmitoylethanolamide published by the group of Professor Costa in 2008 [3]. These data however are not based on studies conducted with PEA-um. The PEA used in the Costa study is explicitly described as unmicronized, pure PEA. Costa et al. describe the PEA they tested as follows:* “PEA was purchased from Cayman Chemical (Ann Arbor, MI, USA), dissolved in ethanol : saline (1 : 9), and used at a dose of 10 mg/kg.” *Cayman only provides laboratories with pure, unformulated PEA (purity > 99%). Nowhere in the Costa (2008) paper do the authors refer to ultramicronized PEA.

Secondly, the authors state the following:* “However, the highly lipophilic PEA crystalline structure has a poor oral adsorption, thus requiring to be micronized and converted into particles with an elevated surface area to volume ratio, in order to enhance its assimilation.”* This claim is not substantiated by any data in man. There are no pharmacokinetic data available of PEA to substantiate this claim, nor are there data available comparing plasma kinetics and dynamics of pure PEA versus ultramicronized PEA in man. Furthermore, all clinical double-blind placebo controlled studies published so far in more than 3000 patients have been conducted with simple PEA formulations. PEA was never tested as an ultramicronized or micronized formulation in such trials. It has recently come to our attention that the most impressive double-blind placebo controlled PEA study in 636 patients suffering from sciatic pain received ethics committee agreement in 1992 and was conducted with LG 2110/1, a code for pure PEA. Although the authors claim PEA has a poor oral absorption, the results of the clinical studies so far prove that, even if true, this is irrelevant as PEA does have significant and clinical relevant effects in simple, nonultramicronized formulations.

As the study of Cocito et al. only reports the short-term follow-up data of a single center open-label study, in the absence of a placebo group, none of the conclusions of the authors are supported by the study. This study only supports the safety of PEA-um, and the safety of PEA in all known formulations has been documented already sufficiently.

Therefore we need to conclude that the entire paper can only be seen as support for the market introduction of ultramicronized PEA, partly based on experiments conducted with unmicronized PEA. And as we pointed out elsewhere, never has there been any clinical trial conducted and published supporting superiority of any PEA formulation over another [4]. On the contrary, all double-blind placebo controlled trials to date have been conducted with unmicronized PEA and are supportive for such pharmaceutical formulation.
